# The Growth Characteristics of Patients with Noonan Syndrome: Results of Three Years of Growth Hormone Treatment: A Nationwide Multicenter Study

**DOI:** 10.4274/jcrpe.3013

**Published:** 2016-09-01

**Authors:** Zeynep Şıklar, Mikayir Genens, Şükran Poyrazoğlu, Firdevs Baş, Feyza Darendeliler, Rüveyde Bundak, Zehra Aycan, Şenay Savaş Erdeve, Semra Çetinkaya, Ayla Güven, Saygın Abalı, Zeynep Atay, Serap Turan, Cengiz Kara, Gülay Can Yılmaz, Nesibe Akyürek, Ayhan Abacı, Gamze Çelmeli, Erkan Sarı, Semih Bolu, Hüseyin Anıl Korkmaz, Enver Şimşek, Gönül Çatlı, Muammer Büyükinan, Atilla Çayır, Olcay Evliyaoğlu, Pınar İşgüven, Tolga Özgen, Nihal Hatipoğlu, Atilla Halil Elhan, Merih Berberoğlu

**Affiliations:** 1 Ankara University Faculty of Medicine, Department of Pediatric Endocrinology, Ankara, Turkey; 2 İstanbul University Istanbul Faculty of Medicine, Department of Pediatric Endocrinology, İstanbul, Turkey; 3 Dr. Sami Ulus Child Health and Disease Training and Research Hospital, Clinic of Pediatric Endocrinology, Ankara, Turkey; 4 Göztepe Training and Research Hospital, Clinic of Pediatric Endocrinology, İstanbul, Turkey; 5 Marmara University Faculty of Medicine, Department of Pediatric Endocrinology, İstanbul, Turkey; 6 Ondokuz Mayıs University Faculty of Medicine, Department of Pediatric Endocrinology, Samsun, Turkey; 7 Konya Training and Research Hospital, Clinic of Pediatric Endocrinology, Konya, Turkey; 8 Dokuz Eylül University Faculty of Medicine, Department of Pediatric Endocrinology, İzmir, Turkey; 9 Akdeniz University Faculty of Medicine, Department of Pediatric Endocrinology, Antalya, Turkey; 10 Gülhane Military Medical Academy, Department of Pediatric Endocrinology, Ankara, Turkey; 11 Düzce University Faculty of Medicine, Department of Pediatric Endocrinology, Düzce, Turkey; 12 Dr. Behçet Uz Children Disease and Surgery Training and Research Hospital, Clinic of Pediatric Endocrinology, İzmir, Turkey; 13 Osmangazi University Faculty of Medicine, Department of Pediatric Endocrinology, Eskişehir, Turkey; 14 Tepecik Training and Research Hospital, Clinic of Pediatric Endocrinology, İzmir, Turkey; 15 Erzurum Regional Training and Research Hospital, Clinic of Pediatric Endocrinology, Erzurum, Turkey; 16 İstanbul University Cerrahpaşa Faculty of Medicine, Department of Pediatric Endocrinology, İstanbul, Turkey; 17 Sakarya University Faculty of Medicine, Department of Pediatric Endocrinology, Sakarya, Turkey; 18 Bezmialem University Faculty of Medicine, Department of Pediatric Endocrinology, İstanbul, Turkey; 19 Erciyes University Faculty of Medicine, Department of Pediatric Endocrinology, Kayseri, Turkey; 20 Ankara University Faculty of Medicine, Department of Biostatistics, Ankara, Turkey

**Keywords:** Noonan syndrome, growth hormone treatment, growth

## Abstract

**Objective:**

Noonan syndrome (NS) is a multisystem disorder, and short stature is its most striking manifestation. Optimal growth hormone (GH) treatment for NS is still controversial. In this study, using a nationwide registration system, we aimed to evaluate the growth characteristics and the clinical features of NS patients in Turkey and their growth response to GH treatment.

**Methods:**

Children and adolescents with a diagnosis of NS were included inthe study. Laboratory assessment including standard GH stimulation test results were evaluated. Height increment of patients with or without GH treatment were analyzed after three years of therapy.

**Results:**

A total of 124 NS patients from different centers were entered in the web-based system. Short stature and typical face appearance were the most frequently encountered diagnostic features of our patients. Of the 84 patients who were followed long-term, 47 hadreceived recombinant human GH (rhGH). In this group of 47 patients, height standard deviation score (HSDS) increased from -3.62±1.14 to -2.85±0.96 after three years of therapy, indicating significant differences from the patients who did not receive GH treatment. PTPN11 gene was analyzed in 61 patients, and 64% of these patients were found to have a mutation. HSDS at admission was similar in patients with or without PTPN11 gene mutation.

**Conclusion:**

A diagnosis of NS should be kept in mind in all patients with short stature showing systemic clinical findings. GH therapy is effective for improvement of short stature especially in the first two years of treatment. Further studies are needed for optimisation of GH therapy and evaluation of final height data in NS patients.

**WHAT IS ALREADY KNOWN ON THIS TOPIC?**

Noonan syndrome is a multisystem disorders, and short stature is a frequent finding. There are no national data about the clinical and growth characteristics of Noonan syndrome patients until now.

**WHAT THIS STUDY ADDS?**

This study aimed to descript the characteristics of Noonan syndrome at a national level.

## INTRODUCTION

Noonan syndrome (NS) is a genetic, multisystem disorder with variable phenotype. The estimated prevalence of this syndrome was reported to vary between 1 in 1000 and 1 in 2500 live births (1,2). The main clinical characteristics of the syndrome consist of short stature, cardiovascular abnormalities (pulmonary valve stenosis, hypertrophic cardiomyopathy), cryptorchidism, and facial dysmorphology (hypertelorism, ptosis, low-set and posteriorly rotated ears, webbed neck) (3,4,5).

Short stature is a common manifestation of NS (6). Although birthweight and body length are usually normal in NS, pubertal growth is often delayed. At pubertal ages, short stature can be the most striking finding (3). Adult height in these patients is approximately 2 standard deviation (SD) below the mean for healthy adults (7). The causes of the growth disturbances in NS are multifactorial. Growth hormone (GH) deficiency, GH insensitivity, and neurosecretory dysfunction have been reported in these patients (3,8).

While the etiology of short stature is not definitely known, treatment of GH has been shown to improve growth rates (7). The response to GH therapy in NS can be affected by a number of factors such as dose of recombinant human GH (rhGH) and type of genetic mutation. The optimal GH treatment for NS is still controversial.

Genetic mutations associated with NS are involved in intracellular RAS/MAPK signal transduction pathway, leading to dysregulation (7). Until now, eight genes in the RAS/MAPK pathway, namely PTPN11, SOS1, KRAS, NRAS, RAF1, BRAF, SHOC2, and CBL, have been reported to cause NS. The PTPN11 gene encodes the protein SHP2 which is responsible for controlling several developmental processes (3). Several studies have shown that almost half of NS patients had mutations in PTPN11 gene (9,10).

Information about the clinical characteristics, especially in growth parameters of NS patients at national level is scarce. In this study, using a nationwide registration system, we aimed to evaluate the growth characteristics, clinical features, and response to GH treatment of NS patients in Turkey.

## METHODS

In this study, we retrospectively analysed the data of 124 children and adolescents with NS who were being followed in 20 centers in Turkey. Study approval was given by the Ankara University Ethics Committee. A nation-wide web based system was used for data collection which was realized between 15 May 2014 and 15 May 2015. A case recording form which covered demographic, clinical, and laboratory findings of patients was created and uploaded to the web site of FAVOR Web Registry System^®^. Data of patients aged between 0.2-18 years were entered in each center.

Children and adolescents with clinical (based on van der Burgt criteria) and/or genetic diagnosis of NS were included in the study. The following data on the patients’ admission characteristics and clinical findings were collected: short stature, typical face dysmorphology, cardiac (pulmonary stenosis, hypertrophic cardiomyopathy, secundum atrial septal defect (ASD), electrocardiogram abnormalities, atrioventricular canal defect, mitral valve abnormalities, aortic coarctation), chest (pectus carinatum/excavatum, increased internipple distance, scoliosis), renal (unilateral kidney, pelvic dilatation), gastrointestinal (gastroesophageal reflux, recurrent vomiting, hepatomegaly, splenomegaly, feeding difficulties), hematologic (coagulopathy, thrombocytopenia, thrombocyte dysfunction), ocular (strabismus, refractive errors, amblyopia, nystagmus, cataract, fundal changes) anomalies, undescended testes, neuromotor problems (cognitive disorders, learning difficulties, mental retardation). The researchers were also asked to enter other clinical features that were not included in the questionnaire form to the system.

Clinical characteristics including birth weight, height, weight, height SD score (HSDS), body mass index (BMI), bone age at diagnosis were also recorded.

According to van der Burgt criteria, definitive NS was diagnosed by: 1) typical face dysmorphology plus one other major signs, or 2) suggestive face dysmorphology plus two other major or three minor signs. Major signs included 1) typical facial dysmorphology, 2) cardiac findings (pulmonary valve stenosis, hypertrophic obstructive cardiomyopathy, and/or echocardiography typical for NS), 3) height below 3rd centile, 4) chest wall deformities (pectus carinatum, pectus excavatum), 5) positive family history of a first-degree relative with a definite diagnosis of NS, 6) other findings (mental retardation, cryptorchidism, lymphatic dysplasia, etc.). Minor signs included 1) suggestive facial dysmorphology, 2) cardiac defect other than major cardiac signs, 3) height below 10th centile, 4) broad thorax, 5) family history for first-degree relative with suggestive NS, 6) other findings (mental retardation, cryptorchidism, or lymphatic dysplasia) (11).

The questionnaire form also included the pathological genetic test results of the patient, if the genes PTPN11, SOS1, RAF1, KRAS, NRAS, etc. were analysed.

The researchers were also asked to enter to the system laboratory assessments including hormonal [thyroxine, thyroid-stimulating hormone (TSH), insulin-like growth factor 1 (IGF1), IGF-binding protein 3 (IGFBP3)] test results, GH stimulation tests, and bone age. If there were any other pathological laboratory findings, the participating centers were also asked to enter them. The assessment methods of serum IGF1, IGFBP3, and GH were answered optionally. Laboratory assessments included serum GH (ng/mL) levels measured by chemiluminescence method in all centers. Serum IGF1 and IGFBP3 were assayed by immunochemiluminescence (mostly) and immunoradiometric analysis (two cases).

All centers used two pharmacological GH stimulation tests for diagnosis of GH deficiency, in addition to clinical and laboratory characteristics. Sufficient GH response to GH stimulation tests were accepted as a peak GH level of >10 ng/mL. The participating centers were also asked if they had performed IGF generation tests in patients with low growth velocity.

All centers were also asked to enter to the system the yearly height increment of patients who were given GH treatment and those who were not. The follow-up form also included bone age increment and additional features developing during the follow-up.

Entering additional information not included in the questionnaire form was optional.

Statistical analyses were performed by using SPSS for Windows version 22.0 statistical software. Frequencies and percentages represented the descriptive statistics for categorical variables, and mean ± SD values were used for continuous variables. Student’s t, chi-square, and Fisher exact tests were used. For evaluation of long-term growth parameters in groups, repeated measures ANOVA and Bonferroni test for pairwise comparisons were used.

## RESULTS

### Baseline Characteristics

Data of a total of 124 patients with NS (84 males, 40 females) were entered to the web-based system. On admission, the mean age of patients was 8.36±4.5 years, HSDS was -3.13±1.31, and parentally adjusted height deficit was -2.25±1.73 (Table 1). Ninety eight of cases (79%) were prepubertal.

The most frequently seen clinical findings were short stature (88.7%) and typical facial features (88.7%). Cryptorchidism (uni- or bilateral) had been detected in 64% of male patients and cardiovascular anomalies in 62.8% of patients (Table 2).

### Laboratory Characteristic

All NS patients were reported to be euthyroid on admission.

Genetic analysis of PTPN11 gene could be performed in 61 cases, and 39 of these (64%) had mutations. The other mutations that caused NS could not be studied. One patient was reported to have SOS1 gene mutation. Clinical characteristics of patient with and without PTPN11 gene mutation were compared. The only difference detected between the groups was the percentage of typical facial dysmorphology. Patients with PTPN11 gene mutation had 97.4% typical facial dysmorphology, but in patients without PTPN11 gene mutation, this percentage was 78.9% (p=0.036). The remaining characteristics were similar between the two groups (Table 3).

### Growth Hormone Treatment and Long Term Follow-Up

GH stimulation test was performed on 78 patients, and 50 of these showed suboptimal GH response (peak GH below 10 ng/mL). There were no statistically differences at admission in HSDS, serum IGF1 SD, and IGFBP3 SD between patients with GH deficiency and patients without GH deficiency.

Long-term growth follow-up data of 84 patients were evaluated. There were no differences in clinical characteristics on admission (typical face, cardiac, chest, eye, gastrointestinal, and other clinical findings) between these cases and those without follow-up. Among them, 47 patients had been receiving rhGH (mean dose= 0.25±0.05 mg/kg/week). Clinical and laboratory characteristics except HSDS were not different in patients receiving GH therapy as compared to patients not receiving GH therapy. GH therapy was introduced to shorter NS patients (Table 4).

**Figure 1:**
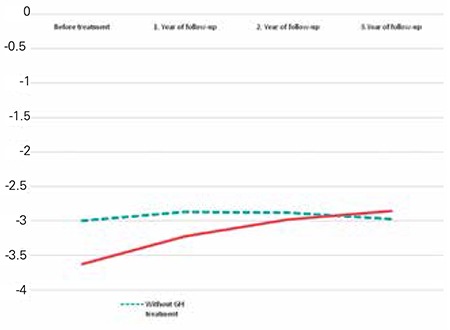
Changes in height standard deviation of patients during follow-up. GH: growth hormone

HSDS increased from -3.62±1.14 to -2.85±0.96 after three years of therapy (Figure 1). Significant differences were observed compared to non-GH-treated patients for each year of therapy (p=0.02) (Table 5). Although bone age increment was evaluated as 1.3 years/year during the first year of therapy, there was no bone age acceleration during the second and third years of therapy.

Some additional findings developed in some patients during the follow-up period. Transient thrombocytopenia and splenomegaly developed in one case. In another case non-ossifying fibroma was diagnosed after beginning GH treatment. Insulin resistance was detected in one case which was not given GH treatment.

Only a small number of cases (n=5) reached their final height. Thus, this report does not cover any findings on final height.

## DISCUSSION

A total of 124 patients (84 males, 40 females) with NS had been registered to the system. All these patients were included in the study. Short stature and typical facial dysmorphism were the most frequent features of this group of patients.

The facial dysmorphology associated with NS such as hypertelorism, epicanthic folds and downward slanting palpebral fissures, low-set posteriorly rotated ears with a thick helix, high arched palate, micrognathia, and a short neck with excess nuchal skin and a low posterior hairline are the most recognizable features. The facial features were indeed the most frequently encountered findings in a high percentage (88.7%) of our patients. The diagnosis of NS is based on clinical features. Patients without typical facial features can easily go unnoticed and therefore, an awareness of suspicion of NS should be increased. In addition, it should be noted that the typical facial features decrease with age and patients with subtle phenotype cango undiagnosed, especially at older ages (11).

NS is one of the most common syndromic causes of congenital heart disease (1,5). In a large cohort study, cardiovascular disease was seen in 81% of NS patients (12). In our series, the proportion of cardiovascular was 62.9%. Pulmonary valve stenosis was detected in 45 patients and was the most frequent heart defect. Hypertrophic obstructive cardiomyopathy (HOCM) is not a frequent finding. van der Burgt (11) indicated that 20% of their NS patients had HOCM. The frequency of partial atrioventricular canal defect, secundum ASD was low in our cases. Anomalies on electrocardiogram such as wide QRS complexes, left axis deviation, giant Q waves were detected in 8 of our 124 patients.

Chest deformities which are characteristics for NS are pectus carinatum and pectus excavatum (11). We found that pectus carinatum occurred at almost twice the rate of pectus excavatum in our patients (32 vs. 17; 25% vs. 13.7%, respectively). The incidence of scoliosis was found lower than those reported in the literature (11,13).

Cryptorchidism is a common problem in male patients with NS. The incidence was reported to be as high as 80% (3,11). In our cases, the percentage of cryptorchidism was 64.2% which is relatively lower than the other series.

Feeding difficulties, especially during infancy, is another problem that can lead to calorie deficiency and growth failure. Gastroesophageal reflux is also a common problem in NS (1,5). The incidence of gastrointestinal problems was 15.3% in our series. In addition, rare gastrointestinal problems including splenomegaly, increase in liver enzyme levels, cholelithiasis, cleft lip and palate were also reported.

Neuromotor disorders, especially mental retardation and learning difficulties, were observed in an important percentage of our patients (Table 1). Mental retardation is usually mild, and this finding is in agreement with the experience of others (11).

Strabismus, refractory errors, amblyopia were the most prominent eye features of our patients. Two of our cases had retinitis pigmentosa.

Hematological and renal system involvements were the least frequently reported findings in our series.

Overall, the systemic findings in our series of patients were not strictly similar to previously reported data. There were some differences either in percentage or in severity of clinical findings. These differences can possibly be attributed to factors such as population characteristics, number of evaluated cases, variation in genetic mutations. We collected data from only pediatric endocrinology clinics. Patient characteristics could be different in those admitted to cardiology or other clinics.

### Genetics

Genetic heterogeneity is a well-known characteristic of NS. There are eight genes in the RAS/MAPK signaling pathway causing NS (3,5). In our series, 61 patients were analysed for PTPN11 gene mutation. The other genes, except for one patient with SOS1 mutation, were not analysed. The rate of PTPN11 gene mutation was 64% of PTPN11 in the patients who underwent genetic analysis.

In the literature, PTPN11-which encodes the protein SHP2- is the most frequently encountered gene associated with NS (3,5,9). Similar to our series, PTPN11 gene mutation was reported in almost half of the NS patients in other studies (2,3,10). SOS1 and RAF1 genes each contribute by 10% to genetic causes of NS, while KRAS and NRAS gene mutations have been detected only in a very small number of cases (3).

There are reports that indicate some differences in NS patients carrying PTPN11 gene mutation from the others (3,9), while others have reported that there were no clinical and laboratory differences between the groups (2,14). Binder et al (10) reported that mutation in PTPN11 gene is associated with mild GH resistance in NS. They emphasized that the mean change in HSDS after one year of GH therapy was lower in mutation-positive patients than mutation-negative patients. In addition, pulmonary stenosis and ASD were most frequently seen in PTPN11 gene mutation-positive patients.

In NS patients with PTPN11 gene mutations, bleeding diathesis, juvenile myelomonocytic leukemia, cardiac defects, typical facies, cryptorchidism, and short stature were also reported to be more common than in patients without PTPN11 gene mutations (3,9,10).

In our series, typical facial features were more prominent in patients with PTPN11 gene mutations, and this was the only difference between patients with and without PTPN11 gene mutation. Growth parameters were similar in mutation-negative and mutation-positive patients.

### Growth Hormone Therapy

The causes of growth deficiency are heterogeneous in NS. GH deficiency, neurosecretory dysfunction, GH insensitivity were suggested factors (8,10,15). In our series, GH stimulation tests were done in 78 patients, and 50 of these (64%) showed suboptimal responses. IGF generation test was performed in two patients, and the results were found to be in line with low GH secretion. Interestingly, the degree of short stature was not different between patients with normal and low GH responses to stimulation tests. Serum IGF1 and IGFBP3 levels were also similar in these two groups of patients.

Some authors suggested that patients with PTPN11 gene mutations often have short stature, low IGF1, and normal or high GH serum levels (10,16). The effect of PTPN11 gene mutations on growth retardation could not be demonstrated in this present study. There were no differences in HSDS between patients with PTPN11 gene mutation and those without mutation. There were also no differences according to serum IGF1 and IGFBP3 levels.

An important fraction of NS patients in this study (n=84) were followed for three years. Forty seven of these were given GH treatment, while 37 were not. HSDS values at admission were lower in GH-treated NS patients, and this finding was the only difference between the GH-treated patients and the non-GH-treated group.

HSDS of NS patients significantly increased during GH treatment, and a positive effect on growth was observed. HSDS increasedby 0.4±0.44 SD during the first year of treatment, and total increments were 0.75±0.55 SD and 0.76±0.41 SD at the end of the second and third years of treatment. HSDS of patients without GH therapy did not change during follow-up (Figure 1).

Despite a positive increment in HSDS noted especially during the first year of GH therapy, this was below 0.5 SD height, a figure which is a relevant indicator of good response to GH therapy (17).

Similar to our study, short-term studies with GH treatment reported an increase of mean HSDS and/or height velocity. After one year of GH therapy, the increment of HSDS was found to be between 0.3 and 0.8 SD. It was also reported that the changes in HSDS and/or height velocity in NS were similar to those observed in Turner syndrome patients (6,18,19,20,21,22). In these studies, the doses of GH were also similar. In our patients, the doses of rhGH varied between 0.2 to 0.35 mg/kg/week, with a mean dose of 0.25 mg/kg/week, which is lower than the offered doses in NS patients. GH resistance in some NS cases would require higher doses of GH.

All these findings indicate that in Turkey, there is still a need to optimise GH therapy in NS patients and most importantly, individualisation of treatment can lead to optimisation of therapy.

No serious side effects related to GH treatment were detected in our series. Transient thrombocytopenia and splenomegaly in one case, non-ossifying fibroma in another case were seen while receiving GH treatment. Insulin resistance was reported in one case who was not receiving GH therapy.

We carefully evaluated the possibility of bone age acceleration in GH-treated NS patients. With GH therapy, an acceleration of bone age was reported in most cases. Bone age has been reported to advance by 1.1-1.2 years/year during GH therapy (15,21,23). We observed a similar trend, especially during the first year of therapy. Actually, NS patients generally have a delayed bone age at the start of GH treatment. It is accepted that the acceleration of bone age reflects normalisation (7).

There are reports indicating that final height of NS patients receiving GH showed a gain of SDS ranging from 0.6 to 1.7 (6,24). We have only a few patients who reached their adult height (n=5) and therefore we cannot report any conclusions.

There are some limitations of our study. First of all, the data was web based and, being from different centers, was very heterogeneous in some clinical or laboratory characteristics. Indications for and dose of GH therapy were not uniform. In addition, the phenotypic, laboratory, and molecular characteristics of NS patients were not similar. The systemic features of NS were more frequently reported in some centers. Response to GH therapy also showed variations, indicating a need for individualisation of GH therapy for optimal response.

In conclusion, in this study, we attempted to demonstrate the clinical and biochemical characteristics of NS patients at a national level. Our data support the findings in previous reports and indicate that in NS patients, GH therapy is useful to improve height deficit, at least during the first two years of therapy. We suggest that GH therapy optimisation is needed for NS patients. Further randomized, observational studies are necessary on patients receiving different GH doses. Also, genetic analysis of patients without PTPN11 gene mutation will provide additional information on NS patients.

## Ethics

Ethics Committee Approval: Ankara University Ethical Committee 2014, approval number: 10.02.2014, 03-84-14, Informed Consent: Retrospective study.

Peer-review: Externally peer-reviewed.

## Figures and Tables

**Table 1:**
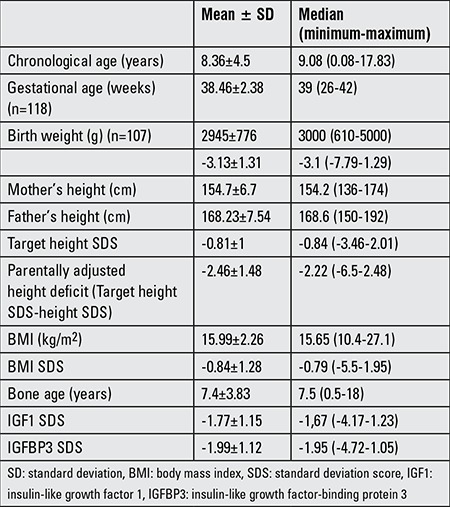
Characteristics of the patients at admission

**Table 2:**
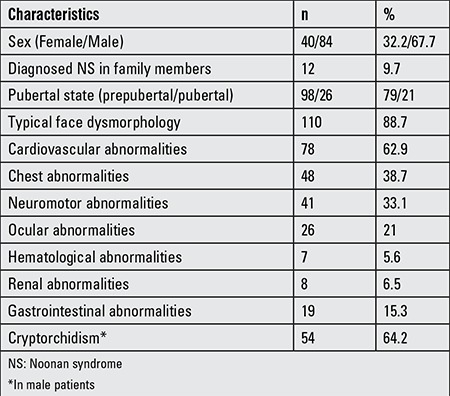
Clinical findings of the patients

**Table 3:**
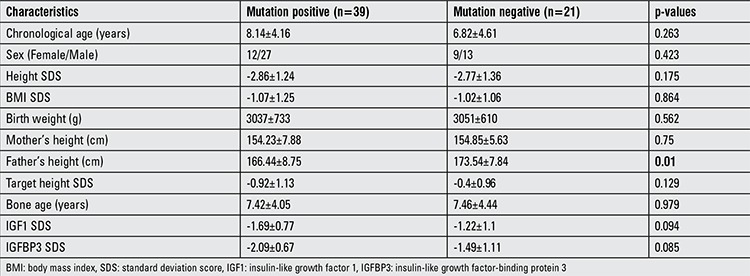
Characteristics of PTPN11 mutation-positive and negative cases

**Table 4:**
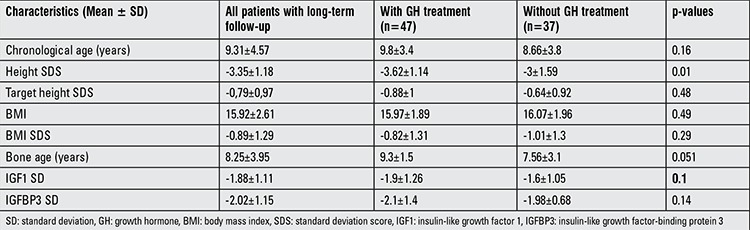
Characteristics of patients with long-term follow-up

**Table 5:**
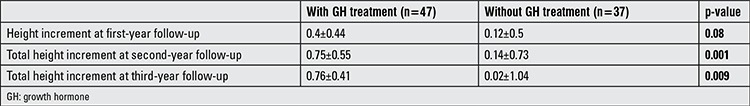
Height increment of patients with Noonan syndrome
